# Temperature Insensitivity Polarization-Controlled Orbital Angular Momentum Mode Converter Based on an LPFG Induced in Four-Mode Fiber

**DOI:** 10.3390/s18061766

**Published:** 2018-06-01

**Authors:** Shen Liu, Yan Zhang, Cailing Fu, Zhiyong Bai, Ziliang Li, Changrui Liao, Ying Wang, Jun He, Yu Liu, Yiping Wang

**Affiliations:** 1 Key Laboratory of Optoelectronic Devices and Systems of Ministry of Education and Guangdong Province, College of Optoelectronic Engineering, Shenzhen University, Shenzhen 518060, China; liushen.szu@gmail.com (S.L.); zhangyan_szu@163.com (Y.Z.); fucailing89@163.com (C.F.); baizhiyong@szu.edu.cn (Z.B.); liziliang2016@email.szu.edu.cn (Z.L.); cliao@szu.edu.cn (C.L.); yingwang@szu.edu.cn (Y.W.); hejun07@szu.edu.cn (J.H.); 2 School of Optoelectronic Engineering, Chongqing University of Posts and Telecommunications, Chongqing 400065, China

**Keywords:** fiber sensor, long period fiber grating, CO_2_ laser micromachining, temperature sensitivity, few-mode fiber

## Abstract

We propose a novel method for generating ±1-order orbital angular momentum (OAM) in long-period fiber gratings (LPFGs) by adjusting a polarization controller (PC). An LPFG, inscribed in a four-mode fiber (4MF) using a CO_2_ laser, was used to generate OAM_±1_ across a broad range of wavelengths from 1530 nm to 1630 nm. Additionally, the OAM vortex phase remained stable while the temperature increased from 23 °C to 50 °C. The LPFG, as a temperature sensor, and its temperature sensitivity was measured to be 38.6 ± 0.37 pm/°C at the resonant wavelength of 1625 nm. This design offers simple fabrication and several properties which are highly beneficial for all-fiber optical communications based on the OAM mode-division multiplexing technique.

## 1. Introduction

Conventional methods for encoding information in optical fiber systems utilize multiplexing techniques via wavelength, amplitude, phase, and polarization of light. However, those techniques were rapidly reaching intrinsic limits due to nonlinear effects. Light beams with orbital angular momentum (OAM) exhibit increased fiber information capacity and offer an exciting new communication modality. Light carrying OAM includes a helical phase in the electric field, proportional to e^−ilφ^, where l is a topological charge and φ is the azimuthal angle. OAM has been extensively studied since its initial realization by Allen et al. in 1992 [1]. Subsequent classical and quantum communication experiments have demonstrated the potential applications of OAM modes for free space communication [2,3,4,5].

Several existing techniques for generating OAM modes require precise optical components, such as a spiral phase plate [6], cylindrical lens mode converter [7], spatial light modulator (SLM) [8,9], or q-plates [10]. This can be disadvantageous for optical communications due to the number of separation components, complex system design, and large free-space-coupling loss. In contrast, all-fiber OAM mode generators have a simpler design and offer several other advantages, such as easy system integration. They are also robust, cost-effective, and do not require precise optical calibration. The use of all-fiber based OAM components has been increasing rapidly and various mode converters have been proposed using fiber gratings. For example, Li et al. [11] converted LP_01_ core modes to an OAM_±1_ mode in a two-mode fiber (2MF) by utilizing a parallel metal slab and long-period mechanical gratings. Li et al. [12] generated OAM_±1_ modes by superposing multiple LP_11_ modes with a micro-phase difference distribution, produced by twisting a few-mode fiber long period grating (FMF-LPG). Zhang et al. [13] demonstrated a method for generating OAM_±1_ modes via an acoustically-induced fiber grating (AIFG) driven by a radio frequency source. However, these techniques required external devices to generate OAM and the experimental designs were difficult to couple or control flexibly. Recently, a novel OAM mode generator was proposed based on a Helical-LPFG inscribed in a standard SMF [14]. This helical-LPFG was inscribed by twisting a standard SMF during hydrogen-oxygen flame heating and was used to fabricate LPFGs in small numbers with a single twist of the fiber.

In this letter, we propose and demonstrate a method for generating ±1-order orbital angular momentum (OAM) by tuning a polarization controller (PC) to vary the input-light polarization of long-period fiber gratings (LPFGs) in a four-mode fiber (4MF). The four-mode fiber long period grating (4FM-LPFG) exhibit asymmetric refractive index modulation, inscribed by periodically etching grooves on the side of the 4MF using a high-frequency CO_2_ laser beam. These OAM_±1_ modes were generated within a broad wavelength range from 1530 to 1630 nm. The OAM mode was detected using space-free interference between light propagating through the LPFG in the 4MF and a reference beam after a distance of 50 m. The LPFG, as a temperature sensor, has a temperature sensitivity of ~38.6 pm/°C at the resonant wavelength of 1625 nm. OAM temperature characteristics were also investigated and the helical interference pattern exhibited only minor intensity changes with increasing temperature. The polarization-dependent loss (PDL) of the LPFG was also measured.

## 2. Fabrication and Characterization of the FM-LPFG

The 4MF (four-mode step index fiber, YOCC) has a core diameter of 19 µm, a cladding diameter of 125 µm, a cladding index of 1.45601, and a core index of 1.46111. So that the 4MF can support four groups of core modes, including: LP_01_ (${\mathrm{HE}}_{11}^{x}$ and ${\mathrm{HE}}_{11}^{y}$), LP_11_ (${\mathrm{TE}}_{01}$, ${\mathrm{HE}}_{21}^{o}$, ${\mathrm{HE}}_{21}^{e}$ and ${\mathrm{TM}}_{01}$), LP_21_ (${\mathrm{EH}}_{11}^{o}$ and ${\mathrm{EH}}_{11}^{e}$, ${\mathrm{HE}}_{31}^{o}$ and ${\mathrm{HE}}_{31}^{e}$), and LP_02_ (${\mathrm{HE}}_{12}^{o}$ and ${\mathrm{HE}}_{12}^{e}$). In this paper, LP_11_ core modes in the 4MF were used to generate OAM_±1_ by superimposing two vector modes with a phase delay of ±π/2, which is expressed as: ${\mathrm{OAM}}_{\text{±}1}^{\text{±}1}={\mathrm{HE}}_{11}^{e}\pm {\mathrm{iHE}}_{11}^{o}$, ${\mathrm{OAM}}_{\text{±}1}^{\text{±}1}={\mathrm{TM}}_{01}\pm {\mathrm{iTE}}_{01}$.

**Figure 1:**
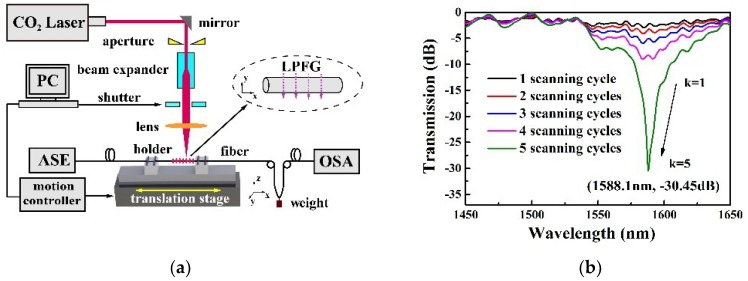
(**a**) Schematic diagram of the CO_2_ laser system employed to induce long-period fiber grating (LPFG) in 4MF; (**b**) Transmission spectrum evolution of a CO_2_-laser-induced LPFG with the scanning cycle (K) increases from 1 to 5.

A schematic diagram of the CO_2_ laser irradiation system, used to inscribe an LPFG on a few-mode fiber, is shown in Figure 1a [15,16,17,18]. The CO_2_ laser beam (SYNRAD 48-1, Synrad, Mukilteo, WA, USA), with a maximum power of 10 W and a high frequency of 5 kHz, was focused onto the 4MF surface using an infrared lens with a focal length of 63.5 mm. As a result, the laser beam spot diameter on the 4MF was only ~50 µm. An electric shutter was used to turn the laser beam on and off. The fabricated 4MF was fixed by a pair of fiber holders connecting to the 2-D translation stage, which was controlled by a computer. During LPFG inscription, one end of the few-mode fiber was fixed to a translational stage fiber holder. The other fiber end was attached to a small weight, with a mass of ~6 g, which provided a constant longitudinal strain along the 4MF fiber and enhanced the efficiency of LPFG inscription. During the whole process of processing, the grating parameters, such as pitch, number of grating periods, and number of scanning cycles, were set through the PC operating interface. Additionally, an amplified spontaneous emission (ASE) light source and an optical spectrum analyzer (OSA) were employed to monitor the transmission spectrum evolution of the LPFG in 4MF. The inscription process of LPFG in 4FM was described, as follows: (1) Scan the 4FM along the vertical (Z) axis orientations with the focused CO_2_ laser beam to create a notch, and then move the fiber along the fiber axis (X) with a distance of one grating pitch; (2) Repeating process (1) for M times (M is the number of grating periods) to accomplish one scanning cycle; (3) Repeating process (2) for K times (K is number of the scanning cycle) until a high-quality LPFG was achieved through repeated scanning.

In our experiment, the 4MF-LPFG exhibited 25 grating periods and a grating pitch of 1200 µm. It has an attenuation of 30.45 dB at the resonant wavelength of 1588.1 nm and the transmission spectrum is shown in Figure 1b. This resonant wavelength displayed a significant blue-shift (toward shorter wavelengths) and resonant dip loss increased with an increasing number of scanning cycles. The 4MF-LPFG transmission spectrum also included several ripples which resulted from mode interference in the 4MF core. Because of the mode mismatch between the 4MF and SMF, a small amount of higher order core modes was excited at the input interface between the SMF and 4MF, and therefore, the mode interference fringes appeared around the resonant wavelength of the 4FM-LPFG [19].

**Figure 2:**
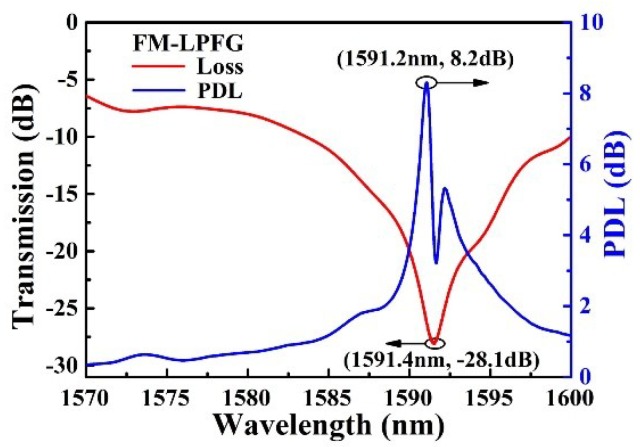
Measured transmission spectrum of the polarization-dependent loss (PDL) for the LPFG.

The polarization characteristics of the 4MF-LPFG were investigated using a tunable laser (Agilent Model 81940A, Agilent Technologies, Santa Clara, CA, USA) and an optical power meter (Agilent Model N7744A, Agilent Technologies, Santa Clara, CA, USA). Polarization-dependent loss (PDL) for LPFG samples was measured at the resonant wavelength of 1591.4 nm. A maximum attenuation of −28 dB was observed as shown in Figure 2. Additionally, the maximum PDL was measured at 8.2 dB near this resonant wavelength. These results are indicative of the asymmetric azimuthal profile for refractive index modulation in the 4FM, inscribed using a focused CO_2_ laser on one side of the optical fiber.

**Figure 3:**
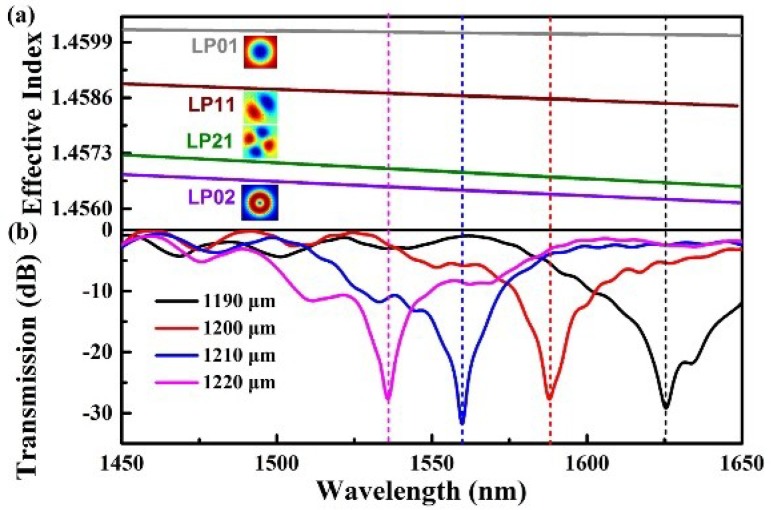
(**a**) Calculated effective refractive indices plotted as a function of wavelength for the LP_01_, LP_11_, LP_21_, and LP_02_ modes; (**b**) The transmission spectra of four CO_2_-laser-induced LPFGs with grating pitches of 1190, 1200, 1210, and 1220 μm.

In order to generate core mode coupling from LP_01_ to LP_11_, the LPFG grating period was first calculated by a finite element analysis software. The results of this numerical simulation (i.e., the core modes n_eff_ of LP_01_, LP_11_, and LP_21_) were shown in Figure 3a. Additionally, the LPFG phase-matching condition could be calculated using the following formula:

λ_res_ = (n_1_ − n_2_)Λ (1)

where λ_res_ is the resonant wavelength, n_1_ and n_2_ are the effective indices (n_eff_) of two coupled modes, and Λ is the designed grating pitch. The relationship between the calculated grating pitches and resonant wavelength, for mode-coupling between the LP_01_ and LP_11_ core modes, can then be calculated from the LPFG phase-matching conditions and the calculated n_eff_ values in the 4MF. This process is demonstrated in Figure 3a. LPFGs were produced with pitches of 1190, 1200, 1210, and 1220 µm. The corresponding transmission spectra, listed in Figure 3b, for resonant wavelengths of different pitches were 1538.8, 1559.7, 1588.0, and 1625.8 nm, respectively. These results demonstrate that the resonance wavelength shifts toward shorter wavelengths as the grating pitch increases. The LPFG converted a fundamental core mode (LP_01_) to a higher core mode (LP_11_) with a coupling efficiency higher than 98%.

## 3. Experiment Results

**Figure 4:**
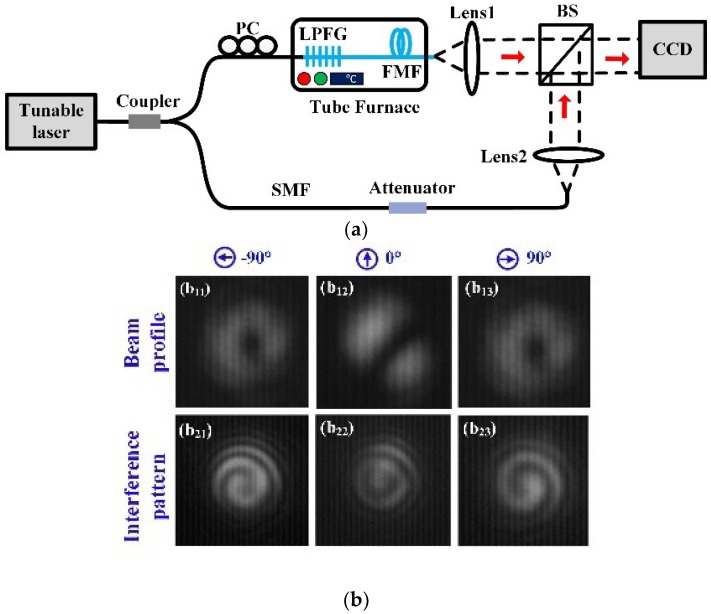
(**a**) Experimental configuration used to detect orbital angular momentum (OAM) modes generated by the CO_2_-laser-induced LPFG; (**b**) (b_11_–b_13_) An experimentally generated vector mode with phase delays of +π/2, 0, and −π/2 and test results are using the same sample with a grating pitch of 1200 µm at 1588.1 nm. (b_21_–b_23_) OAM interference patterns with topological charges of l = +1, l = 0, and l = −1, respectively.

As shown in Figure 4a, an experimental apparatus was developed for measuring OAM modes generated by the 4MF-LPFG. This device was used to confirm the presence of excited OAM_±1_ modes in the LPFG with a grating pitch of 1200 µm and a grating resonant wavelength of 1588.1 nm. The LPFG was placed in a tube furnace with a resolution of 0.01 °C. A tunable laser (Model 81940A, Agilent Technologies, Santa Clara, CA, USA) was utilized with wavelengths ranging from 1520 to 1620 nm. The light beam from this laser was divided into two branches by a fiber coupler (90:10). One part of the light (90%) was propagated into a polarization controller (PC) and an achieved 4MF-LPFG to generate OAM modes, and then collimated into a beam splitter (BS) through a lens (Lens1). Another part of the light (10%) was propagated into an attenuator and then collimated into the BS through another lens (Lens2) as a reference beam. The intensity of the reference beam was controlled to achieve clear interference patterns by the tunable attenuator. The generated OAM modes from the 4MF-LPFG interfered with the reference beam on the BS. The beam profile and interference pattern of the generated OAM can be observed by use of an infrared CCD (Model 7290A, Electrophysics Corp, Fairfield, NJ, USA).

We investigated this pattern for polarization orientations of −90°, 0°, and 90°. As shown in Figure 4(b_11_–b_13_), orientations of −90° and 90° produced a near-infrared mode profile with an annular shape. This occurred in the center at a resonant wavelength of 1588.1 nm when the reference light beam was excluded. Additionally, a polarization orientation of −90° resulted in OAM_+1_ modes, achieved by interference with the reference beam. The corresponding counter-clockwise spiral interference patterns for the OAM_−1_ mode were clearly observed at a resonant wavelength of 1588.1 nm, as shown in Figure 4(b_21_). However, with a polarization orientation of 90° the observed OAM_−1_ mode interference pattern was a clockwise spiral, as shown in Figure 4(b_23_). Near-field output patterns near the resonance wavelength are shown for a polarization orientation of 0° in Figure 4(b_12_). The structure includes a clear two-lobe mode profile, which indicates the LPFG has efficiently converted the LP_01_ core mode to an LP_11_ core mode. The experimental results shown in Figure 4 illustrate that l = ±1 OAM modes were successfully generated at the output end of the LPFG. The fiber can transfer these modes over distances of ~50 m, which could be beneficial for OAM mode division multiplexing in optical communications. The transmission capability of OAM modes for longer distances has been reported. For example, Wang et al. [20] achieved OAM multiplexing transmission with less digital signal processing (DSP) for 8.8 km in a conventional graded-index multi-mode fiber (MMF). Ung et al. [21] demonstrated that the transmission distances of OAM modes was up to 1.1 km by using a large effective index separations few-mode fiber.

This study represents the first time (to our knowledge) that ±1-order OAM modes have been experimentally generated by tuning a polarization controller (PC), placed at the front of 4MF-LPFG an LPFG on a 4FM. The fundamental principle of this OAM conversion can be explained as follows [13,22,23]. The linearly-polarized input light (LP_01_) can be considered the superposition of two vector modes: ${\mathrm{LP}}_{01}^{x}$ and ${\mathrm{LP}}_{01}^{y}$. A phase delay of ±π/2 can be produced between the two components by tuning the PC, converting the linearly-polarized beam to a circularly-polarized ${\mathrm{CP}}^{\text{±}}$ mode. The ${\mathrm{HE}}_{11}^{x/y}$ mode can be selectively converted to ${\mathrm{HE}}_{21}^{e}$ and ${\mathrm{HE}}_{21}^{o}$ modes using the LPFG. Therefore, when left- or right-handed circular polarization modes were input into the 4MF-LPFG, ${\mathrm{CP}}^{\text{±}}$ modes were converted to ${\mathrm{OAM}}_{\text{±}1}^{\text{±}1}={\mathrm{HE}}_{11}^{e}\pm {\mathrm{iHE}}_{11}^{o}$. When the phase delay of two polarization components was 0, the input light was still linearly polarized. As such, there was no spiral phase generation after the light passed through the grating. No spiral phase generation of the output light was observed after coupling of the input light into the LPFG. 

**Figure 5:**
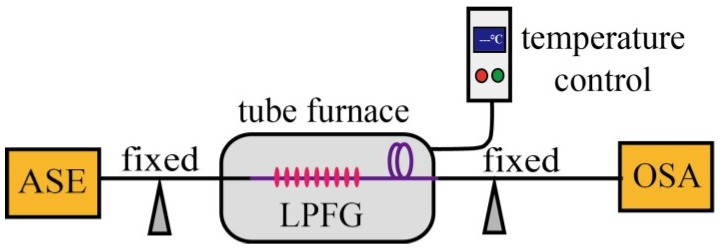
Experiment setup for testing the temperature response of the LPFG.

Experiment setup for testing the temperature response of the LPFG was shown in Figure 5. An LPFG with a pitch of 1200 μm was placed in a tube furnace with a temperature range from room temperature to 100 °C. An ASE light source and an OSA were employed to monitor transmission spectrum evolution of the LPFG during heating. The LPFG sample was heated from 30 °C to 100 °C with a step of 10 °C. After a desired temperature was achieved each time, the temperature was maintained for 20 min, and then the resonant wavelength was recorded. 

**Figure 6:**
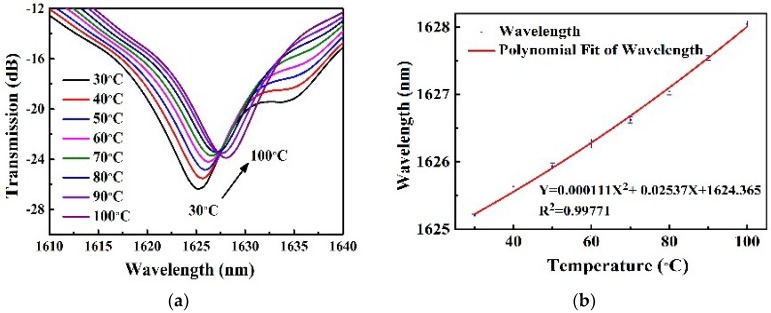
(**a**) Transmission spectra evolution of the 4FM-LPFG with the temperature increased from 30 °C to 100 °C in steps of 10 °C; (**b**) Relationship between the resonant wavelength and the temperature.

As shown in Figure 6a, the resonant wavelength of the LPFG shifted toward the longer wavelength side with the increased temperature from 30 °C to 100 °C. The measured resonant wavelength shift as a function of temperature is shown in Figure 6b, the corresponding temperature sensitivity is about ~38.6 ± 0.37 pm/°C at the resonant wavelength of 1625 nm, and the similar experimental results were also reported by Wang et al. [24]. In this experiment, the measurement error was mainly dependent on the accuracy of the experimental instrument, such as OSA’s resolution of data sampling and the stability of temperature controlled by the tube furnace. In the whole process of the temperature experiment, the OSA with a resolution of 20 pm and the tube furnace with an accuracy of 0.01 °C, were employed, respectively. The corresponding measurement error was calculated to be about ±0.37 pm/°C, where the results of the higher-order nonlinear fitting were considered, as shown in Figure 6b. 

**Figure 7:**
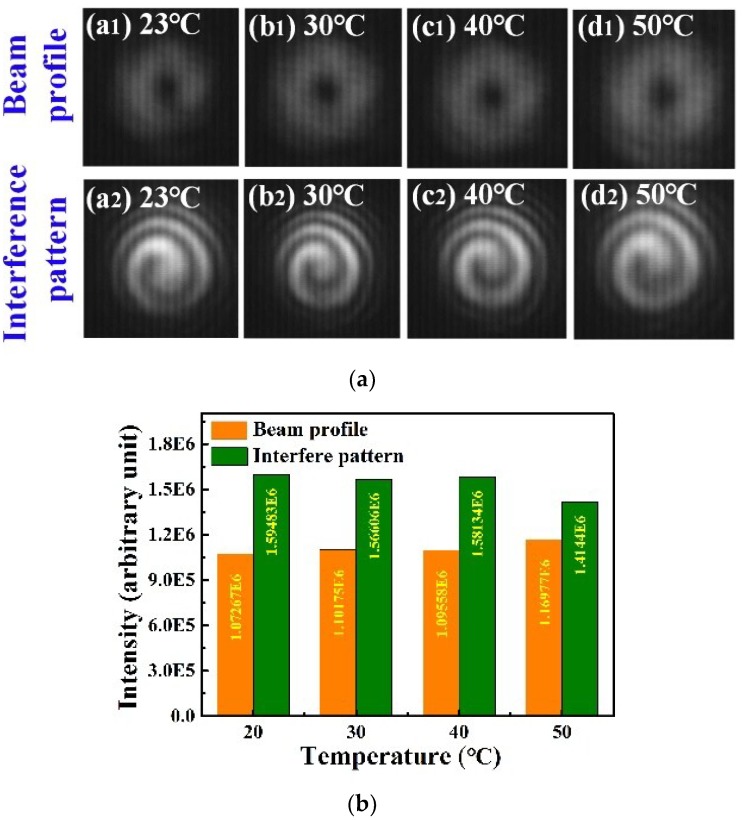
(**a**) Beam profiles and interference patterns of OAM modes generated by the LPFG as the temperature increased from 23 °C to 50 °C; (**b**) The intensity (a. u.), calculated by MATLAB, changed with the temperature increasing from 23 °C to 50 °C.

The temperature characteristics of these OAM modes were also investigated, and the experimental set up was shown in Figure 7a. The 4MF-LPFG was placed in a tube furnace to raise its temperature from 23 °C to 50 °C with a step size of 10 °C. The near-infrared LPFG mode profiles are illustrated without a reference light beam in Figure 7(a_1_–d_1_). The corresponding temperatures were 23 °C, 30 °C, 40 °C, and 50 °C, respectively. Counter-clockwise spiral interference patterns for the OAM_+1_ mode (with a reference beam) are illustrated in Figure 7(a_2_–d_2_), corresponding to the temperatures listed above. It can be seen from Figure 7(a_2_–d_2_) that the helical phase of the OAM remained stable while increasing the temperature from 23 °C to 50 °C. Only minor intensity changes were observed as shown in Figure 7b.

## 4. Conclusions

In conclusion, we have demonstrated an LPFG, based on a 4FM fabricated using a high-frequency CO_2_ laser, with a high conversion efficiency of 98%. OAM_±1_ modes were successfully generated by superimposing two linear polarization modes (${\mathrm{HE}}_{21}^{o}$ and ${\mathrm{HE}}_{21}^{e}$) with a phase delay of ±π/2, achieved by tuning the PC for controlling the input-light polarization of a LPFG. The FM-LPFG, as a temperature sensor, has a temperature sensitivity of ~38.6 pm/°C at the resonant wavelength of 1625 nm. We subsequently tested the effect of temperature characteristics on the LPFG and its effect on propagation of the OAM modes with the temperature increasing. An OAM_±1_ generator of this type has significant potential for enhancing data transmission capacity in all-fiber optical communication systems.
